# YAP-dependent transcriptional responses in tenocytes under cyclic tensile loading: a conditional knockout RNA-sequencing study

**DOI:** 10.3389/fbioe.2026.1901935

**Published:** 2026-10-15

**Authors:** Xuewu Xing, Alexander Piacentini, Yuki Suzuki, Leonardo A. Sanchez, Chris Mendias, Scott A. Rodeo

**Affiliations:** 1 Department of Orthopaedics, Tianjin First Central Hospital, Tianjin, China; 2 Orthopedic Soft Tissue Research Program, Hospital for Special Surgery, New York, NY, United States; 3 Performance Medicine Institute, Phoenix, AZ, United States

**Keywords:** conditional gene knockout, dieerentially expressed genes, mechanotransduction, tendon repair, yes-associated protein

## Abstract

**Background:**

Tendon injuries are prevalent across various populations and significantly impair quality of life, presenting substantial healthcare challenges.

**Purpose:**

This study aimed to characterize Yes-associated protein (YAP) -dependent transcriptional responses in tenocytes under cyclic tensile loading, using a conditional YAP knockout approach combined with RNA sequencing. Given that tenocytes *in vivo* are continuously exposed to mechanical loading, this design was intended to identify genes whose regulation by YAP becomes critical under mechanical challenge.

**Methods:**

Employing conditional gene knockout techniques with RosaCreERT2 YAP-floxed mice and mechanical loading protocols, alongside RNA sequencing and advanced bioinformatics analyses.

**Results:**

We identified 54 differentially expressed genes (DEGs) in tenocytes after YAP knockout, including six upregulated and 48 downregulated genes. Notably, upregulated genes included Gzmc and Gm28151, while downregulated genes included Car3 and Rgs2. Gene Ontology (GO) and Kyoto Encyclopedia of Genes and Genomes (KEGG) enrichment analyses highlighted significant pathways, such as the PPAR signaling pathway, emphasizing their relevance in tendon pathophysiology. Furthermore, CIBERSORTx analysis revealed significant differences in immune cell infiltration, particularly in Treg and CD4 naive T cells, suggesting a link between YAP-dependent pathways and immune responses in tendon healing. Drug prediction analyses identified several small molecules interacting with hub genes, including LTC4S and CXCL13, indicating potential pharmacological targets for therapeutic intervention.

**Conclusion:**

Despite limitations such as a lack of *in vivo* validation and small sample size, this research underscores the pivotal role of YAP in tenocyte mechanotransduction and identifies key DEGs and pathways that may inform future therapeutic strategies for tendon injuries. Future studies should focus on the *in-vivo* implications of these findings and explore the potential for immune modulation and pharmacological interventions in tendon repair.

## Introduction

Tendon injuries represent a significant clinical challenge due to their complex healing processes and the substantial morbidity they can cause across various populations (Millar et al., 2021). The prevalence of these injuries not only impacts the quality of life for affected individuals but also poses a considerable burden on healthcare systems globally. Current research has primarily focused on elucidating the cellular mechanisms underlying tendon healing, emphasizing the roles of various growth factors and cellular responses. However, the specific contributions of mechanotransduction—the process through which cells convert mechanical stimuli into biochemical signals—and its regulatory pathways remain poorly characterized, particularly concerning the involvement of the Yes-associated protein (YAP) signaling pathway (Dupont et al., 2011).

Despite the established importance of mechanotransduction in cellular physiology, significant knowledge gaps remain regarding its specific roles in tendon cell dynamics and healing. Previous studies have elucidated critical functions of YAP in various cellular contexts, including its response to mechanical stress and regulation of gene expression (Codelia et al., 2014; Chen et al., 2024). However, no previous study has examined YAP function specifically in mature tenocytes, nor has the transcriptomic consequence of YAP deletion in this cell type been reported. Consequently, the unanswered biological question is how YAP deletion affects the transcriptomic landscape of mechanically stimulated tenocytes—and which downstream pathways and potential therapeutic targets may be identified. This study directly addresses this gap by characterizing the mechanotransductive responses of tenocytes and the regulatory mechanisms involving YAP.

In musculoskeletal tissues, YAP has been shown to regulate multiple downstream signaling pathways that are critical for tissue homeostasis and repair. For example, YAP modulates PPAR signaling, which governs adipogenic differentiation and metabolic flexibility, thereby influencing the balance between tenogenic and non-tenogenic cell fates. In addition, YAP participates in immune regulation by coordinating the expression of inflammatory cytokines and macrophage polarization, which is essential for proper healing responses. Furthermore, YAP-driven metabolic remodeling—including shifts in glycolysis and oxidative phosphorylation—supports the energetic demands of activated cells during mechanotransduction. These established connections highlight the pleiotropic roles of YAP and provide an important framework for interpreting the transcriptomic changes induced by YAP deletion in tenocytes, particularly in the context of PPAR signaling, immune regulation, and metabolic remodeling.

Immune regulation is a critical yet often overlooked aspect of tenocyte biology. Although tenocytes are the major resident cells in tendon tissue, they do not act in isolation during repair; rather, they communicate with immune cells through paracrine signaling. YAP has been shown to modulate the expression of cytokines and chemokines in stromal cells, thereby influencing the recruitment and polarization of macrophages and other immune cells during tissue repair. In the context of tendon healing, YAP-mediated paracrine signals from tenocytes may help orchestrate the early inflammatory response and subsequent resolution phases, which are essential for effective regeneration. In this study, we performed CIBERSORTx analysis to predict the potential immune microenvironment based on the transcriptomic signatures of isolated tenocytes. Although this approach cannot directly measure immune cell infiltration, it leverages the gene expression patterns of tenocytes to infer the immune landscape that may exist in the *in vivo* tendon environment. This analysis is therefore biologically relevant, as it provides insights into how YAP deletion in tenocytes could alter the immune–tenocyte crosstalk that governs tendon repair.

To address the research questions, this study employs a multifaceted approach utilizing conditional gene knockout techniques in a mouse model, specifically RosaCreERT2 YAP-fl mice. This model allows for precise manipulation of YAP expression in tenocytes, facilitating the investigation of its role under mechanical loading conditions. Moreover, RNA sequencing and comprehensive bioinformatics analyses were employed to analyze gene expression changes in response to YAP knockout and mechanical stimuli (Figure 1). Such an integrative methodology not only enhances the specificity of gene targeting but also provides a comprehensive understanding of the resultant molecular alterations.

**FIGURE 1:**
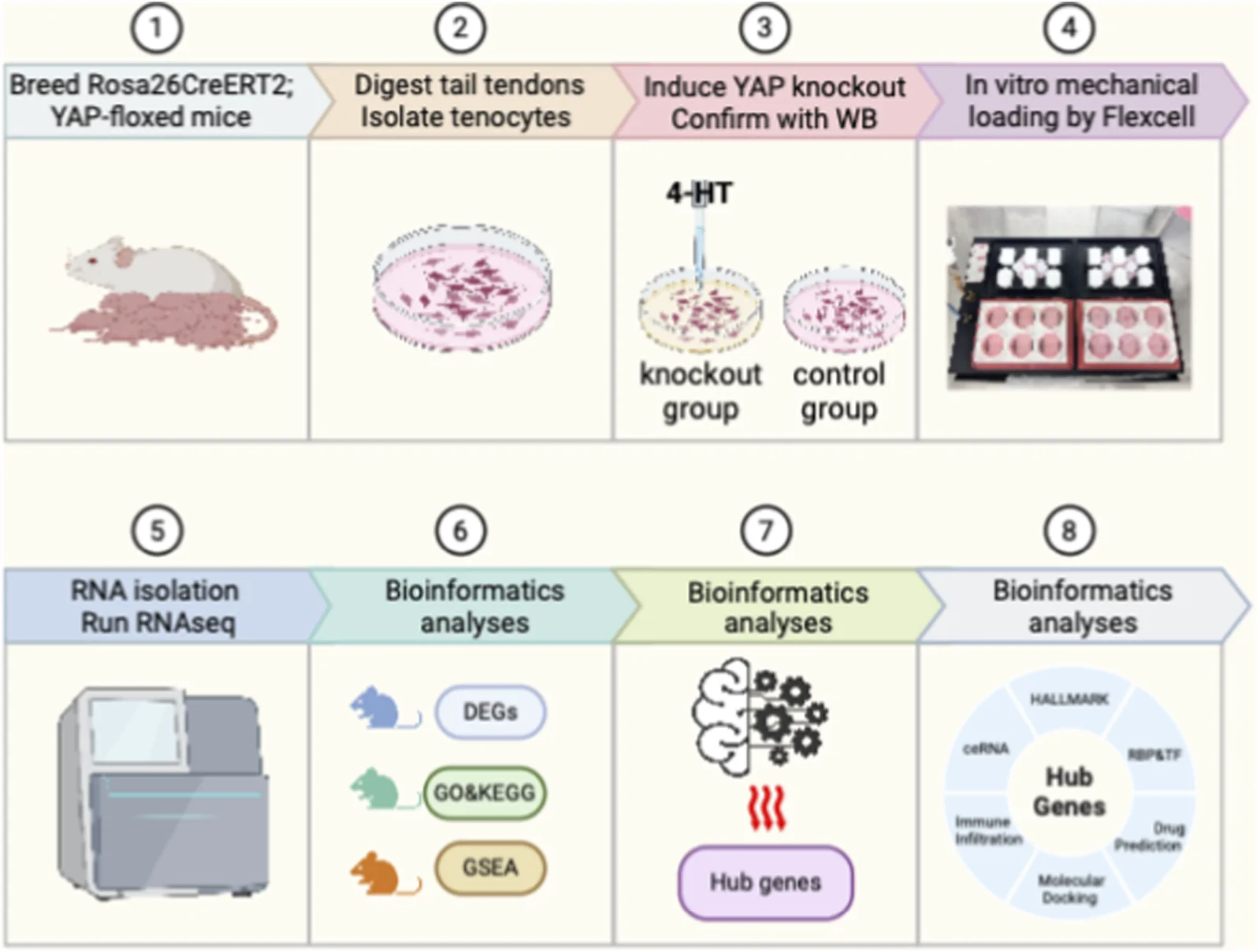
Flowchart of YAP project.

The primary objective of this study was to characterize transcriptomic changes associated with YAP deletion in tenocytes under cyclic tensile loading, and to identify downstream signaling pathways that may be affected by YAP regulation in this mechanical context. Rather than directly addressing tendon healing, we aimed to provide a descriptive transcriptomic resource for tenocyte mechanobiology, with the expectation that these findings will inform future mechanistic studies. We fully acknowledge that the *in vitro* nature of the present model limits direct extrapolation to *in vivo* tendon repair, and we therefore frame the observed gene expression changes as hypothesis-generating. Further studies, including *in vivo* injury models and functional perturbation experiments, will be required to determine whether the YAP-dependent transcriptomic responses identified here have broader relevance to tendon physiology and pathology.

## Materials and methods

### Conditional gene knockout mice, mechanical loading of tenocytes *in vitro*, and RNA sequencing

This study was approved by the IACUC of Weill Cornell Medical College. Rosa26^CreERT2/CreERT2^ mice, in which the tamoxifen-sensitive CreERT2 recombinase enzyme is expressed from the ubiquitous Rosa26 locus (JAX stock 008463) (Ventura et al., 2007), and YAP^flox/flox^ mice in which exons one and two of YAP are flanked by loxP sites (JAX stock 027929) (Zhang et al., 2010), were obtained from the Jackson Laboratory. Genotype of mice was determined by PCR analysis of DNA obtained from tail tendon biopsies. These two strains were backcrossed for several generations to generate R26^CreERT2/CreERT2^:YAP^flox/flox^ mice to allow for the conditional inactivation of YAP in response to treatment with 4-hydroxytamoxifen (4HT) *in vitro*. Cells that were not treated with 4HT are referred to as YAP+, while those that have been treated with 4HT are referred to as YAP^Δ^.

Tail tendons were harvested and digested to isolate tenocytes specifically from 12 3-month-old male mice according to standard protocol (Disser et al., 2022). Tenocytes from six mice were pooled for each experimental group (control and 4-OHT-treated) to obtain sufficient cell numbers for RNA extraction and sequencing, with three biological replicates per group (each replicate derived from two mice). Cells were allowed to grow to passage three (P3) in 5% CO_2_ incubator at 37 °C. After culturing a sufficient number of tenocytes, we induced the knockout of the YAP gene by treating cells *in vitro* with 4-hydroxytamoxifen at a concentration of 1 µM for 48 h. Tenocytes that did not receive tamoxifen served as the control group. YAP knockout was confirmed through Western blot analysis (Supplementary Figure S2), ensuring that the genetic alteration was effectively achieved. Subsequently, the tenocytes were seeded into collagen gels placed in linear troughs in the FlexCell Tissue Train plates (Flexcell International Corporation, Burlington, NC, United States of America) (Li et al., 2023), with a density of 100,000 cells per well, organized according to a specific experimental layout designed to test various conditions.

The Flexcell device was used to apply an *in-vitro* mechanical loading regimen consisting of 4% strain at a frequency of 1 Hz for a continuous period of 24 h. This mechanical stimulation protocol simulates the physiological conditions that tenocytes experience *in vivo*. Following this period of mechanical loading, the cells were harvested for RNA isolation. The isolated RNA samples were then sent to the Weill Cornell Genomics Core, where they underwent analysis of RNA integrity followed by BULK RNAsequencing (NovaSeq X Plus, NEB Ultra II Directional RNA Library Prep, 10B Flow Cell-Paired End Read 2x100 Cycles) to generate comprehensive data on the transcriptomic profile of the tenocytes.

Quality of all RNA and library preparations was evaluated on a TapeStation (Agilent). Sequencing libraries were prepared with NEBNext® Ultra™ II Directional RNA Library Prep and sequenced by Genomics Resource Core Facility at Weill Cornell Medicine. Adapters and low-quality bases were trimmed per lane with fastp (-q 19 -n 20 -L 20). Trimmed reads were aligned per lane with STAR v2.7.7a to the mouse GRCm38/mm10 genome with the GENCODE vM25 annotation. Reads were assigned to genes with featureCounts v2.0.1. Library quality was assessed with fastp, RSeQC, Qualimap RNA-seq QC, Picard duplication metrics and preseq, and aggregated with MultiQC v1.9. Gene-level counts were analyzed in R 4.3.2 with edgeR v4.0.

### Difference analysis

We utilized the R package “edgeR (version 3.36.0)” (Zhou et al., 2014) to identify differentially expressed genes (DEGs) between the control group and the experimental group (YAP knockout) using the count matrix, with screening criteria of |log2Fold Change| >1 and FDR corrected P value < 0.05.

### GO and KEGG pathway enrichment analysis

Gene Ontology (GO) (Gene Ontology consortium: going forward, 2015) enrichment analysis encompasses three categories: Biological Process (BP), Molecular Function (MF), and Cellular Component (CC) analysis. The Kyoto Encyclopedia of Genes and Genomes (KEGG) (Kanehisa and Goto, 2000) is a bioinformatics resource used to mine metabolic pathways that are enriched and significantly altered in gene lists. The R package “clusterProfiler (version 4.2.2)” (Yu et al., 2012) was used to perform GO and KEGG enrichment analysis on the differentially expressed genes in tenocytes before and after YAP knockout (p value < 0.05).

### Gene Set Enrichment Analysis (GSEA)

Gene Set Enrichment Analysis (GSEA) (Subramanian et al., 2005) is an algorithm that determines whether a predefined set of genes shows statistically consistent differences between two biological states. We employed the R package “limma” (version 3.50.0) (Ritchie et al., 2015) to conduct differential expression analysis between the control group and the experimental group (YAP knockout) to determine the fold change (FC) in gene expression between the two groups. GSEA was performed using the R package “clusterProfiler (version 4.2.2)” on an ordered list of all genes arranged by log2FC, with 1000 gene set permutations conducted for each analysis. The mouse-ortholog hallmark gene sets were used as the reference gene set, stored in the Molecular Signatures Database (MSigDB) (Subramanian et al., 2005; Liberzon et al., 2015). Gene sets with P < 0.05 were considered significantly enriched.

### Machine learning

SVM-RFE is a machine learning method that trains subsets of features from different categories to narrow down the feature set and identify the most predictive features. To compute and select linear models while keeping valuable variables, we used the “glmnet” (version 2.25.0) package in R for LASSO regression. Binary distribution variables were then used for LASSO classification, selecting the minimum error to establish the model. This study identified the most significant feature genes as hub genes by integrating the results from both SVM-RFE and LASSO regression methods.

### Immune infiltration

To evaluate whether the cultured tenocyte samples contained any residual non-tenocyte signals, we performed an exploratory analysis using CIBERSORTx (Dapena and Feltner, 1989), a computational method originally developed to estimate immune cell proportions from complex tissues. It should be emphasized that this algorithm was not designed for, and does not provide, quantitative estimates of immune cell abundance in cultured cells. A reference set comprising 25 immune cell subtypes and 511 genes was used to calculate relative enrichment scores from the gene expression profiles of each sample. The resulting scores were visualized using the R package ggplot2 (version 3.3.6) (Ito and Murphy, 2013). These results are presented as a supplementary, exploratory check only and should not be interpreted as quantitative measures of immune infiltration.

### RBP-mRNA network construction

This study employed the open-source platform StarBase (https://starbase.sysu.edu.cn/tutorialAPI.php#RBPTarget) to analyze lncRNA interactions. We used CLIP-seq, degradome-seq, and RNA-RNA interaction data to explore the relationship between mRNA and RNA-binding protein (RBP) expression. For YAP knockout tenocytes, we defined the cutoff criteria for identifying key mRNA-RBP pairs as p < 0.05, with clusterNum ≥5, and clipExpNum ≥5. Subsequently, the RBP-mRNA network was constructed using Cytoscape.

### TF-mRNA network construction

Transcription factors regulate transcription by recognizing specific DNA sequences, playing a role in various complex biological processes. TRRUST (https://www.grnpedia.org/trrust/, Transcriptional Regulatory Relationships Unraveled by Sentence-based Text mining) (Han et al., 2018) is a manually curated transcriptional regulatory network database that contains interaction information between transcription factors and their target genes in humans and mice. This data can be used to predict common transcription factors that regulate key genes in mice.

### ceRNA network construction

To explore the unclear mechanisms of competing endogenous RNA (ceRNA) in tendon injury, we utilized the miRDB (https://mirdb.org/index.html), miRTarBase (https://mirtarbase.cuhk.edu.cn/∼miRTarBase/miRTarBase_2022/php/index.php) (Huang et al., 2022), and starbase2.0 (https://starbase.sysu.edu.cn/starbase2/index.php) (Li et al., 2014) databases to predict the microRNAs associated with key genes, aiming to identify the lncRNAs linked to these common microRNAs and ultimately construct the ceRNA network.

### DGIdb drug prediction

The Drug-Gene Interaction Database (DGIdb, https://dgidb.org) is a public resource that compiles records of genes, drugs, and their interactions to assist clinicians and researchers in generating hypotheses and making discoveries (Cannon et al., 2024). We utilized the DGIdb database to predict potential drugs or small molecules that interact with key genes.

### Molecular docking

To evaluate the binding energy and interaction patterns between candidate drugs/small molecules and their target proteins, we used AutoDockTools-1.5.7, software designed for protein-ligand docking (Angerani et al., 2021). We downloaded the SDF files of the top five drugs from PubChem. These files were converted to mol2 format using OpenBabel (version 2.4.1). We then imported the ligand’s mol2 file into AutoDockTools 1.5.7, set the torsion, and saved it as a pdbqt file. The PDB file of the target protein was downloaded from the PDB database, and the protein was dehydrated and deflected in the molecular visualization tool PyMOL (version 2.5.0). The protein was hydrogenated in AutoDockTools 1.5.7 and output as a pdbqt format file. Molecular docking was performed in AutoDockTools 1.5.7, with the binding energy indicating how likely the receptor and ligand are to bind; lower binding energy indicates higher affinity between the receptor and ligand. The lower the binding energy, the more stable the conformation of the receptor. The results of molecular docking were visualized in PyMOL.

### Statistical analysis

We used R software v4.1.2 for statistical analysis. Spearman correlation tests were used to assess the correlation between two parameters. Wilcoxon tests were used to compare differences between two groups, and Kruskal–Wallis tests were used to compare differences among three or more groups. A two-sided p-value of less than 0.05 was deemed statistically significant.

## Results

### Differential enrichment analysis between YAP knockout and control groups

By comparing YAP knockout and control, a total of 54 differentially expressed genes (DEGs) were identified, with statistically significant differences between the two groups (|log2Fold Change| >1, FDR adjusted P value < 0.05). In the YAP knockout tenocytes samples, six genes were upregulated and 48 genes were downregulated (Supplementary Table S1). All DEGs were visualized using a volcano plot (Figure 2A). Furthermore, a heatmap illustrated the top five upregulated genes (Gzmc, Gm28151, Cdsn, Galnt16, Tnn) and the top five downregulated genes (Car3, Plin4, Hp, Rgs2, Scd1), ranked by P value (Figure 2B).

**FIGURE 2:**
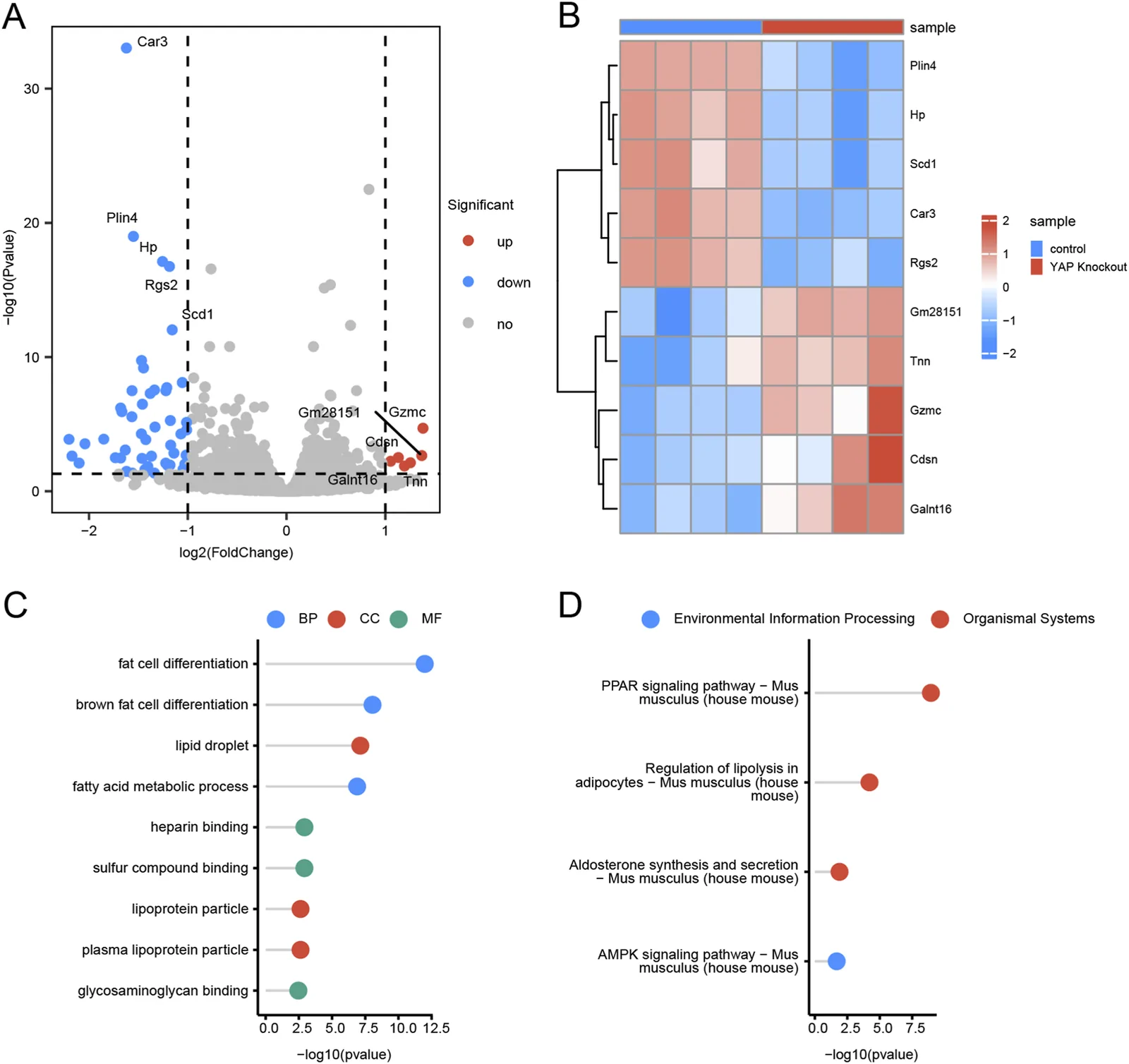
Differential enrichment analysis between YAP knockout and Control groups. **(A)** Volcano plot of DEGs between YAP knockout and Control samples. Red circles represent upregulated genes, blue circles represent downregulated genes, and gray circles represent genes with no significant difference. **(B)** Heatmap of the most significantly different DEGs between YAP knockout and Control samples. **(C)** Lollipop chart showing the GO enrichment analysis results of DEGs. **(D)** Lollipop chart showing the KEGG enrichment analysis results of DEGs.

To further investigate the functions related to DEGs, we performed GO and KEGG enrichment analyses on the 54 DEGs. Figure 2C presents the results of the GO enrichment analysis, which revealed that these genes were significantly enriched in various categories. In biological processes (BP), they were associated with fat cell differentiation, brown fat cell differentiation, and fatty acid metabolic processes. For cellular components (CC), they were linked to lipid droplets, lipoprotein particles, and plasma lipoprotein particles. Lastly, in molecular functions (MF), they were involved in heparin binding, sulfur compound binding, and glycosaminoglycan binding (Supplementary Table S2). The KEGG analysis identified several key enriched pathways, including the PPAR signaling pathway, regulation of lipolysis in adipocytes, aldosterone synthesis and secretion, and AMPK signaling pathway in *Mus musculus* (house mouse) (Figure 2D; Supplementary Table S3).

### GSEA enrichment analysis between YAP knockout and control groups

To better understand the mechanisms of DEGs in YAP knockout *versus* control groups, we performed GSEA enrichment analysis. Using pathway information from the MsigDB database as a reference, we selected the most significant pathways based on normalized enrichment scores (NES) (Supplementary Table S4). The GSEA results showed significant enrichment of the following pathways in the YAP knockout group: ADIPOGENESIS (NES = 2.1902, adjusted P = 0.015, FDR = 0.0082, Figure 3A), CHOLESTEROL HOMEOSTASIS (NES = 2.1269, adjusted P = 0.015, FDR = 0.0082, Figure 3B), EPITHELIAL-MESENCHYMAL TRANSITION (NES = 2.0811, adjusted P = 0.015, FDR = 0.0082, Figure 3C), ANDROGEN RESPONSE (NES = 1.416, adjusted P = 0.027, FDR = 0.0148, Figure 3D), and UV RESPONSE UP (NES = 1.3902, adjusted P = 0.015, FDR = 0.0082, Figure 3E). In contrast, the control group showed significant enrichment of E2F_TARGETS (NES = −1.4807, adjusted P = 0.015, FDR = 0.0082, Figure 3F).

**FIGURE 3:**
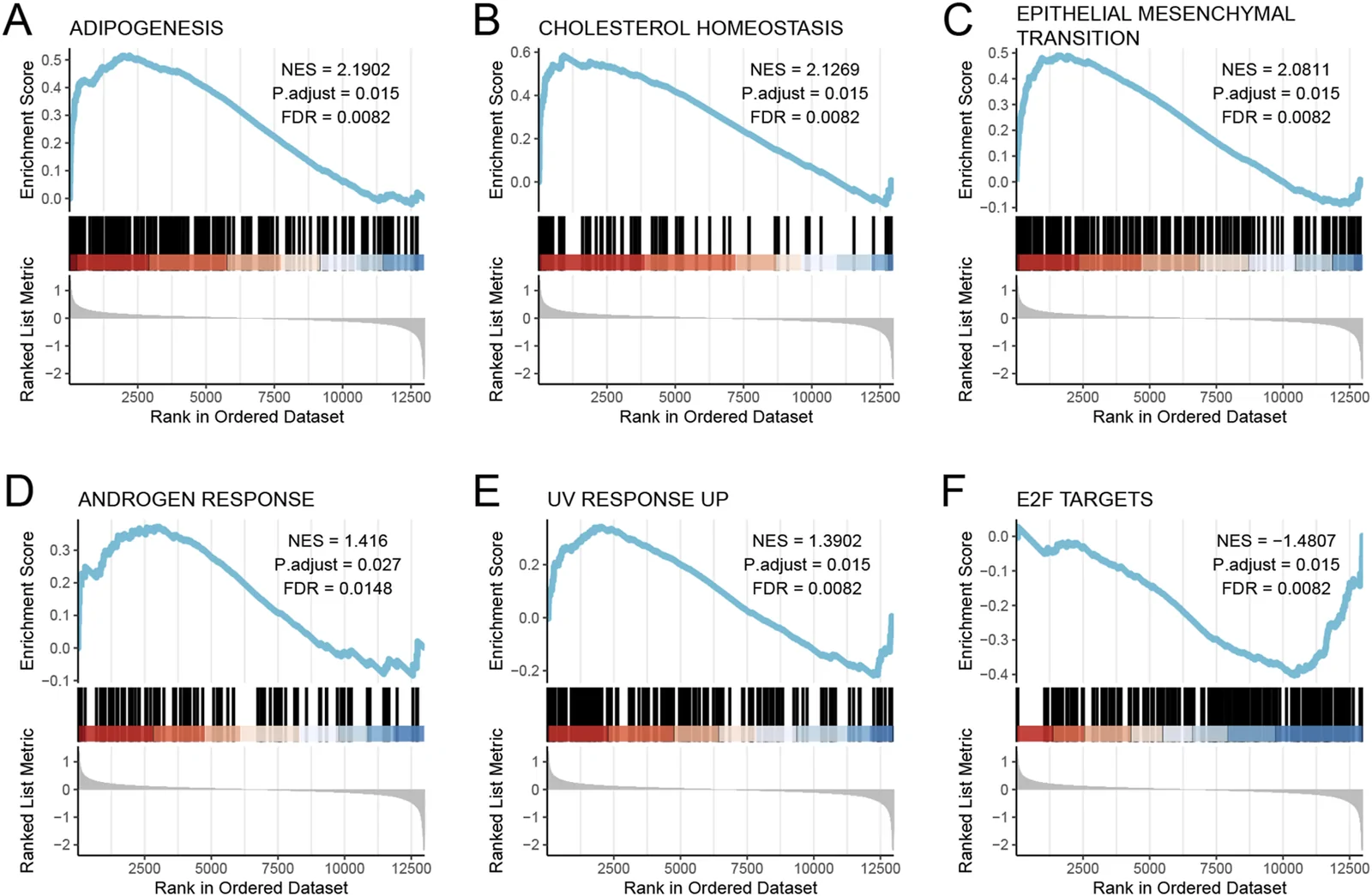
GSEA enrichment analysis between YAP knockout and Control groups. GSEA analysis shows significant enrichment of ADIPOGENESIS **(A)**, CHOLESTEROL_HOMEOSTASIS **(B)**, EPITHELIAL_MESENCHYMAL_TRANSITION **(C)**, ANDROGEN_RESPONSE **(D)**, UV_RESPONSE_UP **(E)**, and E2F_TARGETS **(F)** in both groups.

### Screening hub genes using multiple machine learning algorithms

To prioritize candidate genes among the DEGs for further investigation, we applied LASSO regression and SVM-RFE algorithms. These analyses were employed as a hypothesis-generating approach rather than to construct a predictive classifier. LASSO regression identified seven genes (Figures 4A,B), whereas SVM-RFE identified 39 genes (Figures 4C,D). The intersection of the two methods yielded seven hub genes for subsequent analysis (Figure 4E): Car3, Cxcl13, Hvcn1, Ltc4s, Plin4, Rasd1, and Rgs2. It should be noted, however, that these results were derived from a limited number of biological replicates and should be interpreted with caution; validation in larger cohorts is required to assess their generalizability.

**FIGURE 4:**
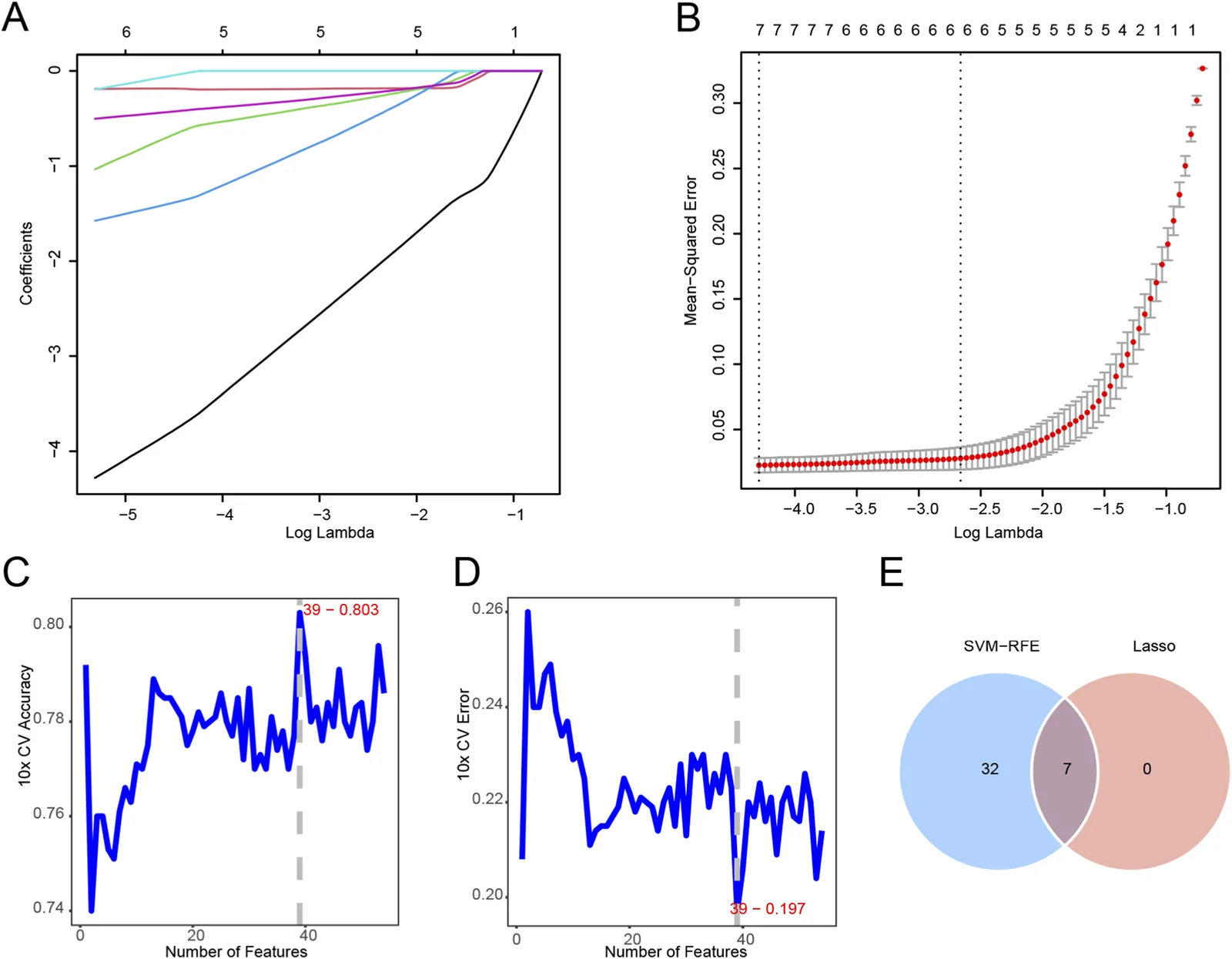
Screening hub genes using machine learning methods. **(A)** The trajectory of independent variable changes in LASSO regression, with the x-axis representing the logarithm of the independent variable lambda and the y-axis representing the coefficients. **(B)** Confidence intervals for each lambda in LASSO regression. **(C)** SVM accuracy curve. **(D)** SVM error rate curve. **(E)** Venn diagram showing the acquisition of hub genes.

### Expression and correlation analysis of hub genes

We examined the expression patterns of seven hub genes in YAP knockout and control groups, as shown in Figure 5A, which highlights significant differences in gene expression between the two groups. We also analyzed the correlations among the seven hub genes, as illustrated in Figure 5B, revealing significant positive relationships. This indicates a strong interactive relationship among the hub genes, suggesting that they may collectively contribute to tendon healing.

**FIGURE 5:**
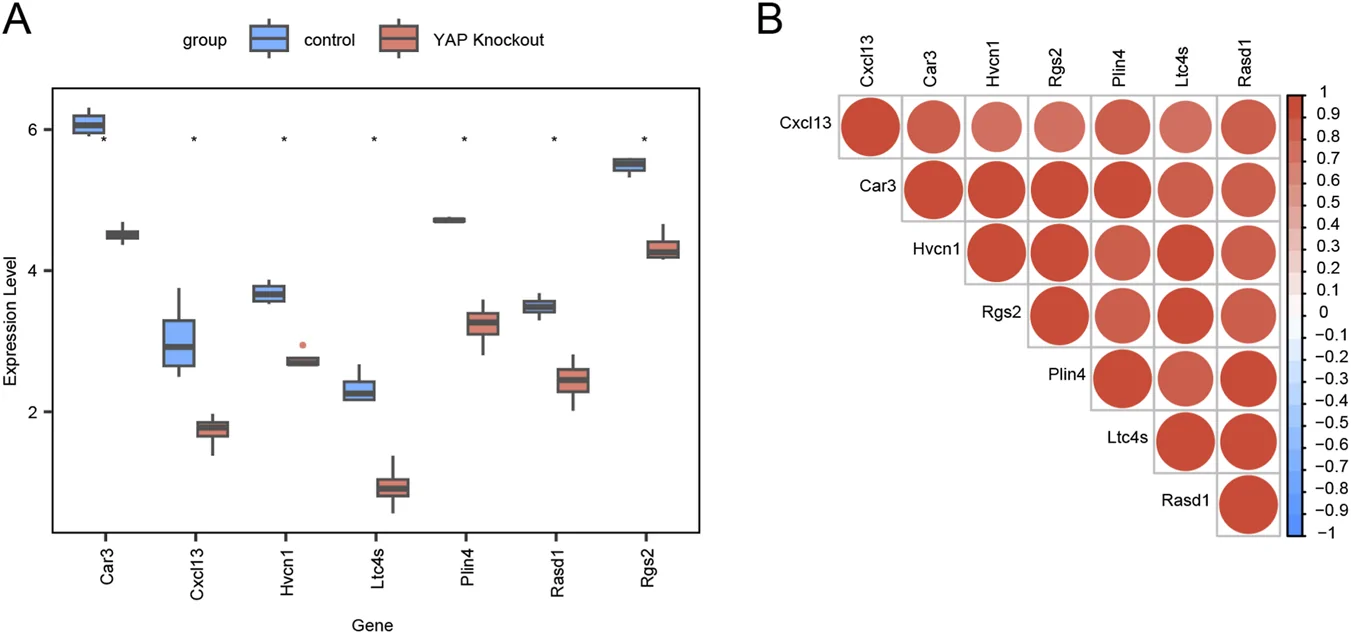
Expression patterns and correlation analysis of hub genes between YAP knockout and Control groups. **(A)** Box plot of expression patterns of hub genes between YAP knockout and Control groups. **(B)** Bubble plot of hub gene correlations. ****p < 0.0001, ***p < 0.001, **p < 0.01, *p < 0.05.

### Immune infiltration analysis

We used the CIBERSORTx method to further investigate the infiltration levels of 25 immune cell types between YAP knockout and control groups. Our analysis revealed that most immune cells exhibited no significant differences between the YAP knockout and control groups (P > 0.05). However, CD4^+^ naive T cells and regulatory T cells (Tregs) showed significant differences (P < 0.05, Figure 6A; Supplementary Table S5). This indicates that these immune cells may be more strongly associated with tendon healing. We also displayed the correlation between immune cells and key genes using a bubble plot, showing that all seven key genes were positively correlated with Tregs (Figure 6B).

**FIGURE 6:**
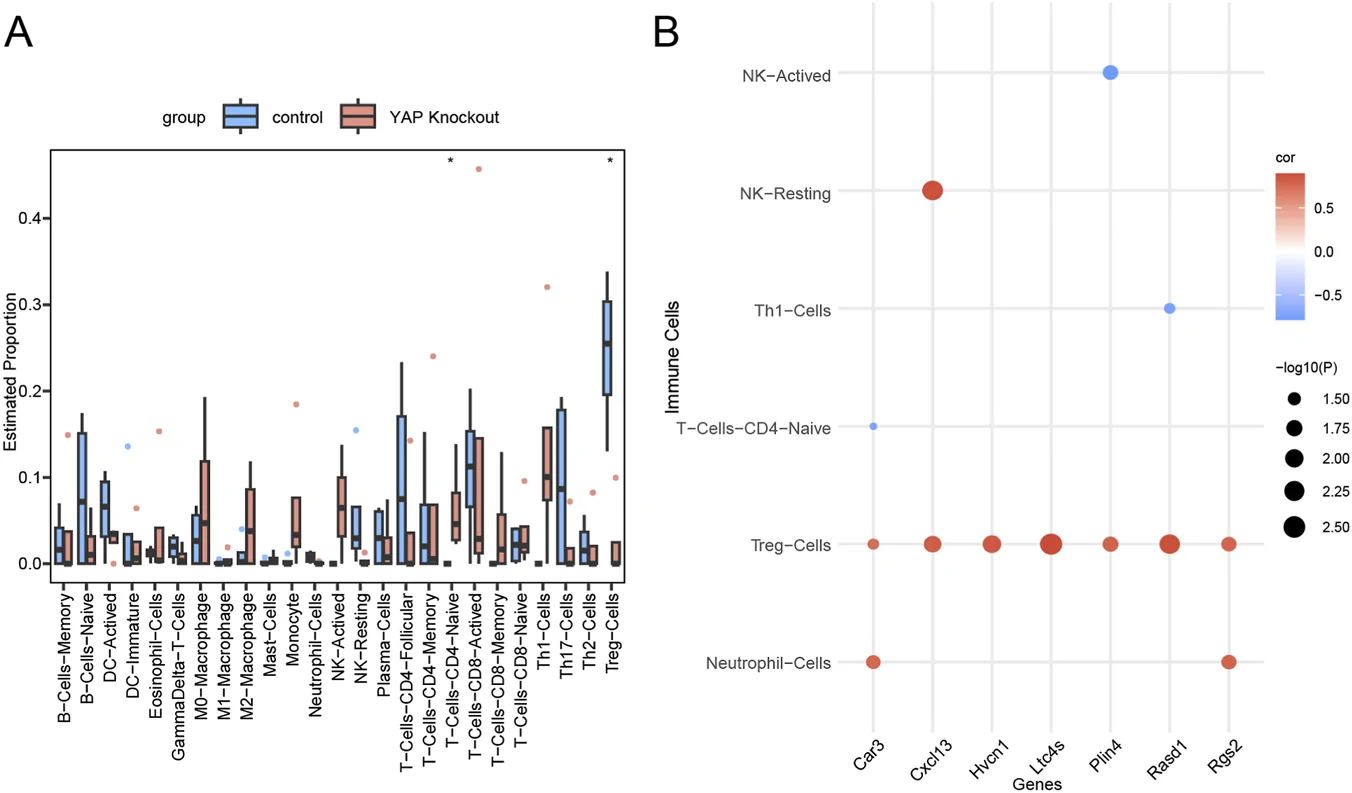
Immune infiltration analysis between YAP knockout and Control groups. **(A)** Estimated immune cell infiltration proportions showing differences between YAP knockout and Control groups. **(B)** Bubble plot showing significant correlations between key genes and immune cells. ****p < 0.0001, ***p < 0.001, **p < 0.01, *p < 0.05.

### HALLMARK pathway enrichment analysis

We used GSVA to analyze differences in 50 HALLMARK signaling pathways between YAP knockout and control groups (Supplementary Table S6) to understand how common pathways relate to disease progression during tendon healing and to examine the correlations with hub genes. Significant differences were observed between the two groups in several pathways, including HALLMARK_UV_RESPONSE_UP, HALLMARK_TNFA_SIGNALING_VIA_NFKB, HALLMARK_P53_PATHWAY, HALLMARK_MTORC1_SIGNALING, HALLMARK_INTERFERON_GAMMA_RESPONSE, HALLMARK_HYPOXIA, HALLMARK_APICAL_SURFACE, HALLMARK_APOPTOSIS, HALLMARK_BILE_ACID_METABOLISM, HALLMARK_ANGIOGENESIS, and HALLMARK_ADIPOGENESIS (Figure 7).

**FIGURE 7:**
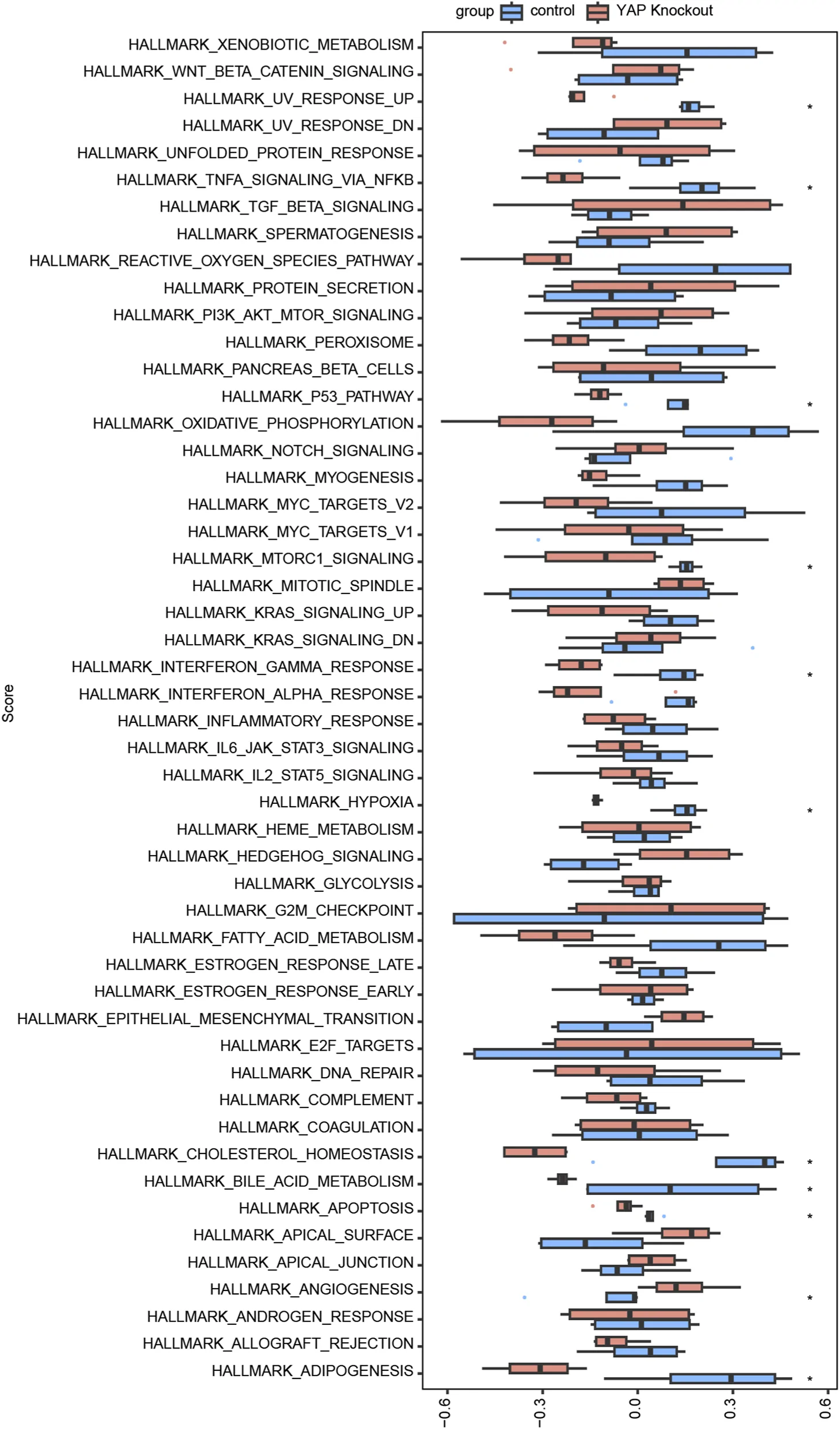
Comparison of 50 HALLMARK signaling pathways between YAP knockout and control groups. ****p < 0.0001, ***p < 0.001, **p < 0.01, *p < 0.05.

In addition, we also explored the correlation analysis between hub genes and signaling pathways (Figure 8). A total of 21 pathways were significantly correlated with the seven hub genes. Among these, Car3 correlated with 18 of the 21 pathways, while Cxcl13 exhibited significant positive correlations with HALLMARK_P53_PATHWAY and HALLMARK_MYC_TARGETS_V2, along with significant negative correlations with HALLMARK_HEDGEHOG_SIGNALING and HALLMARK_EPITHELIAL_MESENCHYMAL_TRANSITION. Hvcn1 showed significant positive correlations with several pathways, including HALLMARK_UV_RESPONSE_UP, HALLMARK_TNFA_SIGNALING_VIA_NFKB, HALLMARK_PEROXISOME, HALLMARK_P53_PATHWAY, HALLMARK_MYOGENESIS, HALLMARK_INTERFERON_GAMMA_RESPONSE, HALLMARK_HYPOXIA, HALLMARK_CHOLESTEROL_HOMEOSTASIS, HALLMARK_APOPTOSIS, and HALLMARK_ADIPOGENESIS. Ltc4s was significantly positively correlated with HALLMARK_UV_RESPONSE_UP, HALLMARK_TNFA_SIGNALING_VIA_NFKB, HALLMARK_INTERFERON_GAMMA_RESPONSE, HALLMARK_HYPOXIA, HALLMARK_CHOLESTEROL_HOMEOSTASIS, and HALLMARK_APOPTOSIS. Plin4 showed significant negative correlations with HALLMARK_APICAL_SURFACE and HALLMARK_HEDGEHOG_SIGNALING. Rasd1 was significantly positively correlated with HALLMARK_UV_RESPONSE_UP, HALLMARK_TNFA_SIGNALING_VIA_NFKB, HALLMARK_OXIDATIVE_PHOSPHORYLATION, HALLMARK_P53_PATHWAY, HALLMARK_MTORC1_SIGNALING, HALLMARK_HYPOXIA, HALLMARK_CHOLESTEROL_HOMEOSTASIS, HALLMARK_APOPTOSIS, and HALLMARK_ADIPOGENESIS. Rgs2 was significantly negatively correlated with HALLMARK_EPITHELIAL_MESENCHYMAL_TRANSITION, HALLMARK_APICAL_SURFACE, and HALLMARK_ANGIOGENESIS. This indicates that hub genes might influence these pathways and contribute to tendon healing.

**FIGURE 8:**
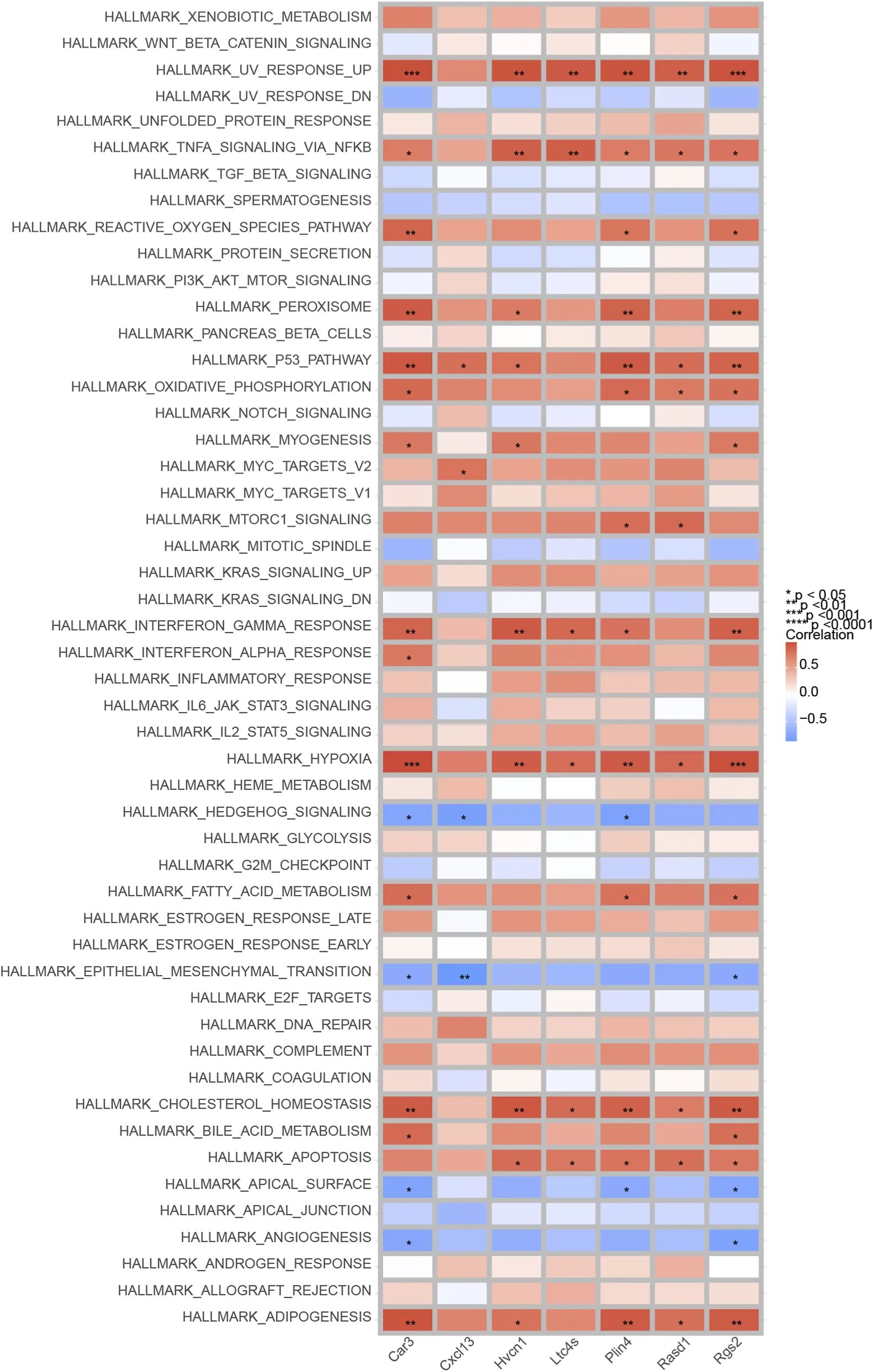
Correlation between hub genes and 50 HALLMARK signaling pathways. ****p < 0.0001, ***p < 0.001, **p < 0.01, *p < 0.05.

### Construction of ceRNA network, RBP network, and TF network for hub genes

To elucidate the potential regulatory mechanisms of the seven hub genes in the YAP knockout tenocytes, we constructed an mRNA–miRNA–lncRNA interaction network (Supplementary Figure S1A; Supplementary Table S7).

Since RNA binding proteins (RBP) interact with mRNA, we utilized the StarBase online database to search and download the corresponding mRNA/RBP pairs for the seven hub mRNAs, of which only Hvcn1, Rgs2, and Plin4 had corresponding RBPs. Using the relationships provided by the online dataset, we constructed an RBP-mRNA network that consists of 11 nodes, including 8 RBPs and three mRNAs, with nine edges (Supplementary Figure S1B; Supplementary Table S8).

Additionally, we analyzed the transcription factor (TF) regulatory network related to the hub genes (Supplementary Figure S1C; Supplementary Table S9).

### Drug prediction and molecular docking for hub genes

The DGldb platform was utilized for drug prediction and molecular docking to identify potential sensitive drugs or molecular compounds related to hub genes. Using DGldb, we conducted a drug sensitivity analysis on seven hub genes and discovered that four of them (LTC4S, CA3, CXCL13, RGS2) were correlated with specific drugs. The drug-gene interaction network (Figure 9A; Supplementary Table S10) illustrates that 12 drugs or molecular compounds had different levels of regulatory impact on the hub genes. We selected the top five small molecular compounds for further analysis and retrieved their two-dimensional structures through PubChem (Figures 9B–F).

**FIGURE 9:**
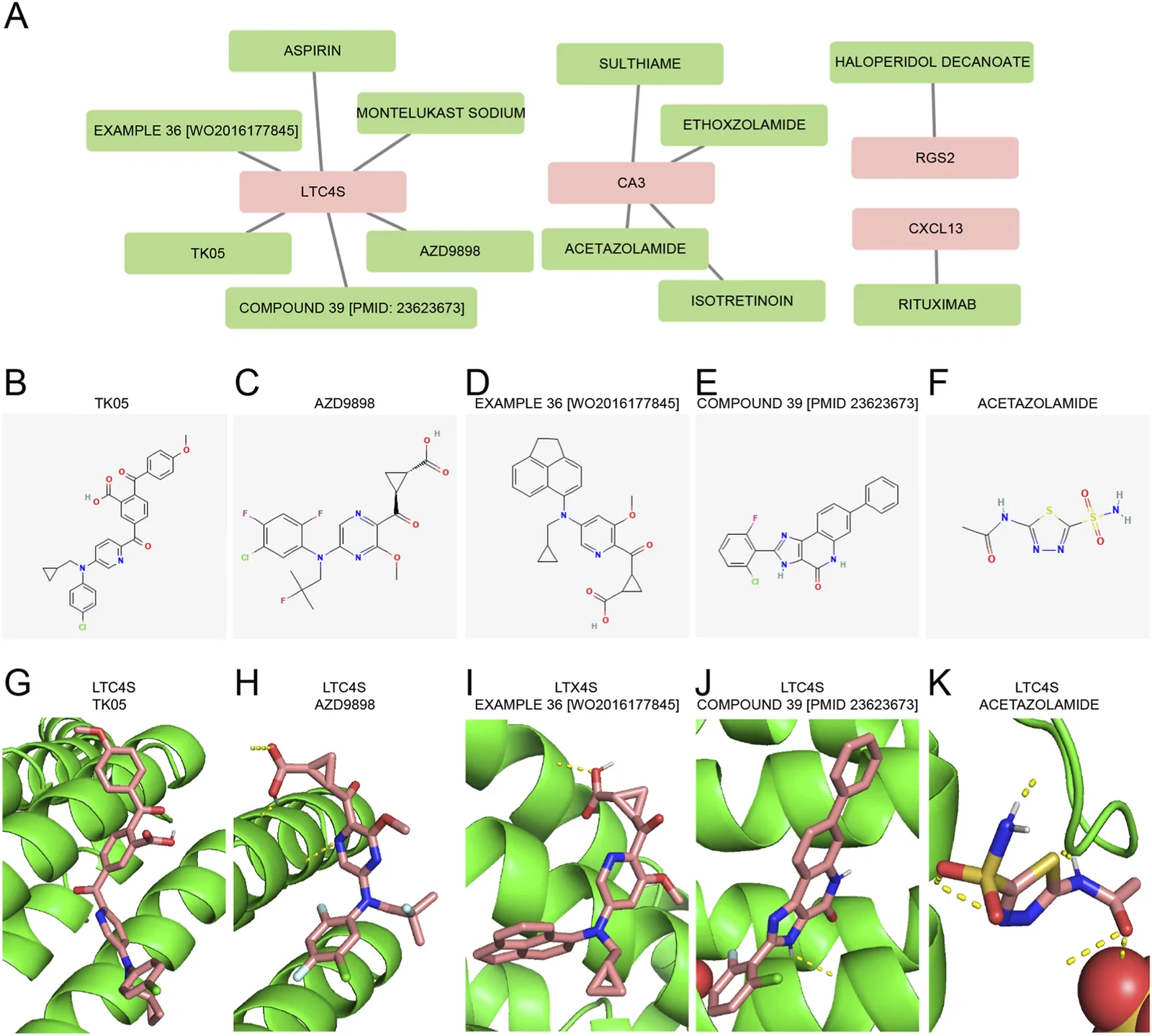
Drug prediction and molecular docking of hub genes. **(A)** mRNA-drugs interaction network of hub genes. Pink rectangular blocks represent hub genes, and green rectangular blocks represent drugs. Two-dimensional structures of TK05 **(B)**, AZD9898 **(C)**, EXAMPLE 36 **(D)**, COMPOUND 39 **(E)**, and ACETAZOLAMIDE **(F)**. Molecular docking results of LTC4S with TK05 **(G)**, AZD9898 **(H)**, EXAMPLE 36 **(I)**, COMPOUND 39 **(J)**, and ACETAZOLAMIDE **(K)**.

Based on the hub gene LTC4S, we performed molecular docking analyses on the small molecular compounds TK05 (Figure 9B), AZD9898 (Figure 9C), EXAMPLE 36 (Figure 9D), COMPOUND 39 (Figure 9E), and ACETAZOLAMIDE (Figure 9F). Molecular docking was performed as an exploratory analysis to examine potential small-molecule interactions involving LTC4S, which was selected because it was among the significant DEGs and functionally linked to the enriched lipid metabolism pathways. A binding-energy threshold of −1.2 kcal/mol was used only as a broad initial filter from the compound library, and all surviving candidates were subsequently ranked by docking score. A positive-control compound was included as a benchmarking reference. The docking results are presented in Table 1 and visualized in Figures 9G–K. It should be emphasized that these results are computational predictions, not evidence of functional binding or therapeutic efficacy. Accordingly, they should be interpreted with caution and require experimental validation before any translational relevance can be considered.

**TABLE 1:** Basic information on candidate drugs and target protein molecular docking.

Molecular name	Targets	Binding energy(kcal/Mol)
TK05	LTC4S	−6.24
AZD9898	LTC4S	−4.8
EXAMPLE 36 (Kleinschmidt et al., 2015)	LTC4S	−5.34
COMPOUND 39 (Shiro et al., 2013)	LTC4S	−7.78
ACETAZOLAMIDE	LTC4S	−4.4

## Discussion

The principal finding of this study is the significant involvement of YAP in modulating gene expression in response to mechanical stimuli, unveiling potential molecular targets for therapeutic intervention in tendon injuries. We identified 54 differentially expressed genes in tenocytes after YAP knockout, including six upregulated and 48 downregulated genes. Upregulated genes included Gzmc and Gm28151, while downregulated genes included Car3 and Rgs2. Gene Ontology (GO) and Kyoto Encyclopedia of Genes and Genomes (KEGG) enrichment analyses highlighted important pathways, such as the PPAR signaling pathway, emphasizing their relevance in tendon pathophysiology. This research not only bridges existing knowledge gaps but also sets the stage for future studies aimed at improving treatment strategies for tendon-related conditions. Mechanical loading was essential to our study because YAP is a key mechanotransducer: its nuclear localization and transcriptional activity are regulated by tensile stimuli and are required for tenocyte homeostasis. By applying mechanical stimulation, we mimicked the native tendon environment and showed that YAP deletion causes significant transcriptomic changes in tenocytes. These results underscore the importance of YAP in converting mechanical signals into downstream cellular responses, and suggest that impaired YAP mechanotransduction may contribute to insufficient tendon repair.

Tendon injuries are a prevalent issue that significantly impacts the quality of life across various populations. These injuries, often resulting from acute trauma or repetitive strain, can lead to chronic conditions such as tendinitis or even complete tendon ruptures, which often require surgery, extensive rehabilitation and may result in long-term disability (Adekanmbi et al., 2017). The intricate healing processes of tendons present substantial challenges, particularly because current treatment options are often limited and can be ineffective (DeFoor et al., 2024). The need for a deeper understanding of the biological mechanisms underlying tendon healing is critical, especially given the role of mechanical stress in tendon homeostasis and the potential for targeted therapeutic interventions.

The results of this study provide significant insights into the molecular mechanisms underlying the response of tendon to mechanical stimulation, particularly the role of mechanotransduction mediated by YAP. The identification of 54 DEGs between YAP knockout and control tenocytes highlights YAP’s critical regulatory function in tenocyte responses to mechanical stimuli. The upregulation of genes such as Gzmc and Gm28151 and downregulation of genes like Car3 and Rgs2 indicate that YAP is intricately involved in modulating the expression of genes essential for tendon cell function and healing processes. This suggests that targeting these DEGs could pave the way for novel therapeutic strategies aimed at enhancing tendon repair mechanisms (Yu et al., 2016). Additionally, understanding how YAP interacts with these genes may shed light on broader cellular responses to mechanical forces, which could have implications for various musculoskeletal disorders and their treatment.

GO and KEGG enrichment analyses of the differentially expressed genes identified several signaling pathways that may be relevant to tenocyte biology. In particular, enrichment of the PPAR signaling pathway was observed, raising the possibility that lipid metabolism and energy homeostasis contribute to the baseline metabolic regulation of tenocytes under mechanical load. It is important to note, however, that these findings were obtained in an *in vitro* tenocyte culture system and are correlative in nature; they do not directly address injury, degeneration, or tendon healing. Accordingly, we refrain from drawing conclusions about therapeutic targets. Future work combining functional perturbation of PPAR signaling with relevant *in vivo* models will be required to assess the biological significance of this pathway. With respect to the individual DEGs, our results are partially consistent with previous reports: Car3 and Rgs2 have been linked to YAP signaling in other systems (Herault et al., 2021). The relationship between PPAR signaling and YAP in tenocytes has not, to our knowledge, been previously reported, and our observation that YAP deletion alters PPAR-related transcription in tenocytes suggests a potentially tendon-relevant regulatory axis that warrants further investigation. Overall, these results support the validity of our approach and underscore the hypothesis-generating value of this transcriptomic resource.

A consistent transcriptional feature of YAP-deficient tenocytes exposed to cyclic tensile loading was the enrichment of lipid metabolism and PPAR signaling pathways. Lipids constitute an important energy source for tenocytes and contribute to membrane organization through effects on fluidity and lipid raft dynamics, processes that can modulate mechanosensitive channels and integrin-mediated signaling. Moreover, lipid-derived mediators are known to influence extracellular matrix remodeling by regulating collagen synthesis, cross-linking, and matrix-degrading enzyme activity. PPARγ, a central regulator of adipogenic differentiation and lipid uptake, has been reported to interact functionally with YAP in diverse cellular contexts. The observed changes in PPAR-related gene expression in YAP-deficient tenocytes raise the possibility that such cross-talk is also operative in tendon cells. However, these findings are based solely on transcriptomic profiling and should be regarded as correlative. Whether the observed metabolic transcriptional program is a cause or consequence of YAP deficiency, and whether it contributes to tenocyte phenotype maintenance, remains to be determined. Future studies combining lipidomic analyses with targeted perturbation of PPAR signaling in tenocytes will be required to establish the functional significance of these pathways. It should also be noted that, because the present experiments were conducted in an *in vitro* tenocyte culture system, the results do not directly inform pathological processes such as fatty infiltration or tendon degeneration, which involve complex tissue-level interactions absent from this model.

Moreover, the findings concerning immune mechanisms, particularly the differential immune cell infiltration observed through CIBERSORTx analysis, raise important considerations regarding the interplay between YAP signaling and immune responses during tendon healing. The significant differences in Treg and CD4 naive T cells infiltration between the YAP knockout and control groups suggest that YAP may modulate immune dynamics in the tendon microenvironment, potentially influencing healing outcomes. This highlights the need for future studies to explore immune modulation as a therapeutic target in tendon injuries, as understanding the immune landscape could lead to more effective strategies for enhancing tendon repair and mitigating chronic inflammation often associated with tendon pathologies (Li et al., 2022). Integrating insights from molecular, signaling, and immune perspectives will be crucial in developing comprehensive strategies for managing tendon injuries and fostering optimal healing processes.

In the context of the current literature, our identified key genes and enriched biological pathways contribute to mechanotransduction in tenocytes through several potential mechanisms. COL1A1 and COL3A1 expression is regulated by mechanical load via the TGF-β/Smad3 pathway, which is a well-established mechanotransduction cascade in tendon cells. ITGA5 (integrin α5) mediates force transmission from the extracellular matrix to the actin cytoskeleton, thereby activating the PI3K-Akt pathway to promote tenogenic gene expression. Additionally, recent studies have highlighted the roles of primary cilia and ion channels such as TRPV4 as additional mechanosensors in tenocytes. Our identified genes may intersect with these pathways, as evidenced by the enrichment of focal adhesion and ECM-receptor interaction pathways in our analysis. These findings collectively suggest that the key genes we identified are integral components of the mechanotransduction network that governs tenocyte function under mechanical loading.

The limitations of this study must be acknowledged, as they could impact the robustness and applicability of the findings. First, the absence of *in vivo* validation restricts the ability to confirm the physiological relevance of the identified differentially expressed genes and pathways. Second limitation of this study is that the key differentially expressed genes identified by transcriptomic analysis have not been experimentally validated. Future studies employing techniques such as qRT-PCR or Western blotting are warranted to confirm these findings and further elucidate the functional roles of YAP in mechanotransduction. Additionally, the relatively small sample size may hinder the statistical power of the analyses, potentially leading to an underestimation or overestimation of the significance of certain findings. We used three biological replicates per group for RNA-seq, which is a standard minimum for this type of analysis and allowed robust detection of differentially expressed genes. However, this sample size may reduce sensitivity to subtle changes and could affect enrichment analyses. Future work with more replicates will help confirm our findings. Furthermore, the lack of clinical correlation data limits the generalizability of the results to human tendon injuries, underscoring the need for future studies to incorporate larger cohorts and clinical samples to substantiate these preliminary findings.

Although mechanical loading was applied to mimic the native tendon environment, cultured tenocytes cannot fully reproduce the complex *in vivo* microenvironment. Specifically, our *in vitro* system lacks the three-dimensional extracellular matrix (ECM) architecture and biomechanical properties of native tendon, which are critical regulators of cell behavior and YAP signaling. In addition, the absence of immune cells, vascular cells, and other stromal components prevents us from directly assessing the cell–cell interactions that are essential for tendon repair; the immune-related signatures we predicted from tenocyte transcriptomics therefore remain indirect. Furthermore, because our model consists of isolated tenocytes, it cannot capture the dynamic events of whole-tissue repair, such as inflammation resolution, neovascularization, or matrix remodeling. Thus, while our findings provide mechanistic insights into YAP-mediated mechanotransduction, they should be interpreted with caution and require validation in more complex systems, such as three-dimensional organoids, co-culture models, or *in vivo* tendon injury models.

In conclusion, this study characterized YAP-dependent transcriptomic responses in tenocytes subjected to cyclic tensile loading and identified differentially expressed genes and enriched pathways—including PPAR/lipid metabolism—that may be regulated by YAP in this mechanical context. These findings provide a transcriptomic resource for tenocyte mechanobiology and generate hypotheses regarding the molecular basis of YAP function in tendon cells. However, it should be emphasized that all analyses are associative and were performed in an *in vitro* culture system without injury or degeneration. The present results therefore do not directly address tendon repair mechanisms and should not be interpreted as immediate therapeutic targets. Future studies using relevant *in vivo* injury models, such as conditional YAP knockout mice, combined with functional validation of candidate genes (e.g., Car3 and Rgs2 by qPCR and Western blot) and targeted perturbation of PPAR/lipid signaling, will be required to establish whether YAP-dependent transcriptional changes contribute to tendon repair and to assess their translational relevance.

## Data Availability

The data presented in the study are deposited in the National Center for Biotechnology Gene Expression Omnibus, https://www.ncbi.nlm.nih.gov/geo/, accession number GSE346125.

## References

[B1] Adekanmbi I. Zargar N. Hulley P. (2017). An *in vitro* scratch tendon tissue injury model: effects of high frequency low magnitude loading. Connect. Tissue Res. 58, 162–171. 10.1080/03008207.2016.1198338 27294971

[B2] Angerani S. Lindberg E. Klena N. Bleck C. K. E. Aumeier C. Winssinger N. (2021). Kinesin-1 activity recorded in living cells with a precipitating dye. Nat. Commun. 12, 1463. 10.1038/s41467-021-21626-1 33674590 PMC7935933

[B3] Cannon M. Stevenson J. Stahl K. Basu R. Coffman A. Kiwala S. (2024). DGIdb 5.0: rebuilding the drug-gene interaction database for precision medicine and drug discovery platforms. Nucleic Acids Research 52, D1227–d1235. 10.1093/nar/gkad1040 37953380 PMC10767982

[B4] Chen M. Liu Y. Zuo M. Guo C. Du Y. Xu H. (2024). NEDD8 enhances Hippo signaling by mediating YAP1 neddylation. J. Biol. Chem. 300, 107512. 10.1016/j.jbc.2024.107512 38960037 PMC11327456

[B5] Codelia V. A. Sun G. Irvine K. D. (2014). Regulation of YAP by mechanical strain through Jnk and Hippo signaling. Curr. Biol. 24, 2012–2017. 10.1016/j.cub.2014.07.034 25127217 PMC4160395

[B6] Dapena J. Feltner M. E. (1989). Influence of the direction of the cable force and of the radius of the hammer path on speed fluctuations during hammer throwing. J. Biomech. 22, 565–575. 10.1016/0021-9290(89)90008-0 2808441

[B7] DeFoor M. T. Cognetti D. J. Yuan T. T. Sheean A. J. (2024). Treatment of Tendon injuries in the servicemember population across the spectrum of pathology: from exosomes to bioinductive scaffolds. Bioeng. (Basel) 11, 158. 10.3390/bioengineering11020158 38391644 PMC10886250

[B8] Disser N. P. Piacentini A. N. De Micheli A. J. Schonk M. M. Yao V. J. H. Deng X. H. (2022). Achilles tendons display region-specific transcriptomic signatures associated with distinct mechanical properties. Am. Journal Sports Medicine 50, 3866–3874. 10.1177/03635465221128589 36305762

[B9] Dupont S. Morsut L. Aragona M. Enzo E. Giulitti S. Cordenonsi M. (2011). Role of YAP/TAZ in mechanotransduction. Nature 474, 179–183. 10.1038/nature10137 21654799

[B10] Gene Ontology consortium: going forward (2015). Nucleic Acids Research 43 D1049–D1056. 25428369 10.1093/nar/gku1179 PMC4383973

[B11] Han H. Cho J. W. Lee S. Yun A. Kim H. Bae D. (2018). TRRUST v2: an expanded reference database of human and mouse transcriptional regulatory interactions. Nucleic Acids Research 46, D380–d386. 10.1093/nar/gkx1013 29087512 PMC5753191

[B12] Herault S. Naser J. Carassiti D. Chooi K. Y. Nikolopoulou R. Font M. L. (2021). Mechanosensitive pathways are regulated by mechanosensitive miRNA clusters in endothelial cells. Biophys. Rev. 13, 787–796. 10.1007/s12551-021-00839-0 34777618 PMC8555030

[B13] Huang H. Y. Lin Y. C. Cui S. Huang Y. Tang Y. Xu J. (2022). miRTarBase update 2022: an informative resource for experimentally validated miRNA-target interactions. Nucleic Acids Research 50, D222–d230. 10.1093/nar/gkab1079 34850920 PMC8728135

[B14] Ito K. Murphy D. (2013). Application of ggplot2 to pharmacometric graphics. CPT Pharmacometrics Syst. Pharmacol. 2, e79. 10.1038/psp.2013.56 24132163 PMC3817376

[B15] Kanehisa M. Goto S. (2000). KEGG: kyoto encyclopedia of genes and genomes. Nucleic Acids Research 28, 27–30. 10.1093/nar/28.1.27 10592173 PMC102409

[B16] Kleinschmidt T. K. Haraldsson M. Basavarajappa D. Lundeberg E. Thulasingam M. Ekoff M. (2015). Tandem benzophenone amino pyridines, potent and selective inhibitors of human leukotriene C4 synthase. J. Pharmacol. Exp. Ther. 355, 108–116. 10.1124/jpet.115.227157 26283693

[B17] Li J. H. Liu S. Zhou H. Qu L. H. Yang J. H. (2014). starBase v2.0: decoding miRNA-ceRNA, miRNA-ncRNA and protein-RNA interaction networks from large-scale CLIP-Seq data. Nucleic Acids Research 42, D92–D97. 10.1093/nar/gkt1248 24297251 PMC3964941

[B18] Li J. Liu C. Kaefer S. Youssef M. Zhao B. (2022). The mechanotransduction channel and organic cation transporter are critical for Cisplatin ototoxicity in Murine hair cells. Front. Mol. Neurosci. 15, 835448. 10.3389/fnmol.2022.835448 35221917 PMC8866953

[B19] Li J. Zhou X. Chen J. Eliasson P. Kingham P. J. Backman L. J. (2023). Secretome from myoblasts statically loaded at low intensity promotes tenocyte proliferation *via* the IGF-1 receptor pathway. Faseb J. 37, e23203. 10.1096/fj.202301097r 37732638

[B20] Liberzon A. Birger C. Thorvaldsdóttir H. Ghandi M. Mesirov J. P. Tamayo P. (2015). The Molecular Signatures database (MSigDB) hallmark gene set collection. Cell Syst. 1, 417–425. 10.1016/j.cels.2015.12.004 26771021 PMC4707969

[B21] Millar N. L. Silbernagel K. G. Thorborg K. Kirwan P. D. Galatz L. M. Abrams G. D. (2021). Tendinopathy. Nat. Rev. Dis. Prim. 7, 1. 10.1038/s41572-020-00234-1 33414454

[B22] Ritchie M. E. Phipson B. Wu D. Hu Y. Law C. W. Shi W. (2015). Limma powers differential expression analyses for RNA-Sequencing and microarray studies. Nucleic Acids Research 43, e47. 10.1093/nar/gkv007 25605792 PMC4402510

[B23] Shiro T. Kakiguchi K. Takahashi H. Nagata H. Tobe M. (2013). 7-Phenyl-imidazoquinolin-4(5H)-one derivatives as selective and orally available mPGES-1 inhibitors. Bioorg Med. Chem. 21, 2868–2878. 10.1016/j.bmc.2013.03.069 23623673

[B24] Subramanian A. Tamayo P. Mootha V. K. Mukherjee S. Ebert B. L. Gillette M. A. (2005). Gene set enrichment analysis: a knowledge-based approach for interpreting genome-wide expression profiles. Proc. Natl. Acad. Sci. U. S. A. 102, 15545–15550. 10.1073/pnas.0506580102 16199517 PMC1239896

[B25] Ventura A. Kirsch D. G. McLaughlin M. E. Tuveson D. A. Grimm J. Lintault L. (2007). Restoration of p53 function leads to tumour regression *in vivo* . Nature 445, 661–665. 10.1038/nature05541 17251932

[B26] Yu G. Wang L. G. Han Y. He Q. Y. (2012). clusterProfiler: an R package for comparing biological themes among gene clusters. Omics 16, 284–287. 10.1089/omi.2011.0118 22455463 PMC3339379

[B27] Yu C. Ji S. Y. Dang Y. J. Sha Q. Q. Yuan Y. F. Zhou J. J. (2016). Oocyte-expressed yes-associated protein is a key activator of the early zygotic genome in mouse. Cell Res. 26, 275–287. 10.1038/cr.2016.20 26902285 PMC4783469

[B28] Zhang N. Bai H. David K. K. Dong J. Zheng Y. Cai J. (2010). The Merlin/NF2 tumor suppressor functions through the YAP oncoprotein to regulate tissue homeostasis in mammals. Dev. Cell 19, 27–38. 10.1016/j.devcel.2010.06.015 20643348 PMC2925178

[B29] Zhou X. Lindsay H. Robinson M. D. (2014). Robustly detecting differential expression in RNA sequencing data using observation weights. Nucleic Acids Research 42, e91. 10.1093/nar/gku310 24753412 PMC4066750

