# Supplementary material for: YAP-dependent transcriptional responses in tenocytes under cyclic tensile loading: a conditional knockout RNA-sequencing study
Source: Front Bioeng Biotechnol. 2026 Oct 15;14:1901935. doi: 10.3389/fbioe.2026.1901935 (PMC13716730; doi:10.3389/fbioe.2026.1901935)
Supplement: Supplementary file 1 [file Supplementaryfile1.docx]

Figure S1 ceRNA network, RBP network, and TF network of hub genes. (A) ceRNA network. Purple represent lncRNA, pink represent mRNA, and blue represent miRNA. (B) RBP-mRNA regulatory network. Blue represent RBP, and purple represent mRNA. (C) TF network. Green represent transcription factors (TF), and orange represent mRNA.
