# Supplementary material for: YAP-dependent transcriptional responses in tenocytes under cyclic tensile loading: a conditional knockout RNA-sequencing study
Source: Front Bioeng Biotechnol. 2026 Oct 15;14:1901935. doi: 10.3389/fbioe.2026.1901935 (PMC13716730; doi:10.3389/fbioe.2026.1901935)
Supplement: Supplementary file 2 [file Supplementaryfile2.docx]

Figure S2 quantification of YAP protein levels in control and YAP cKO tenocytes
